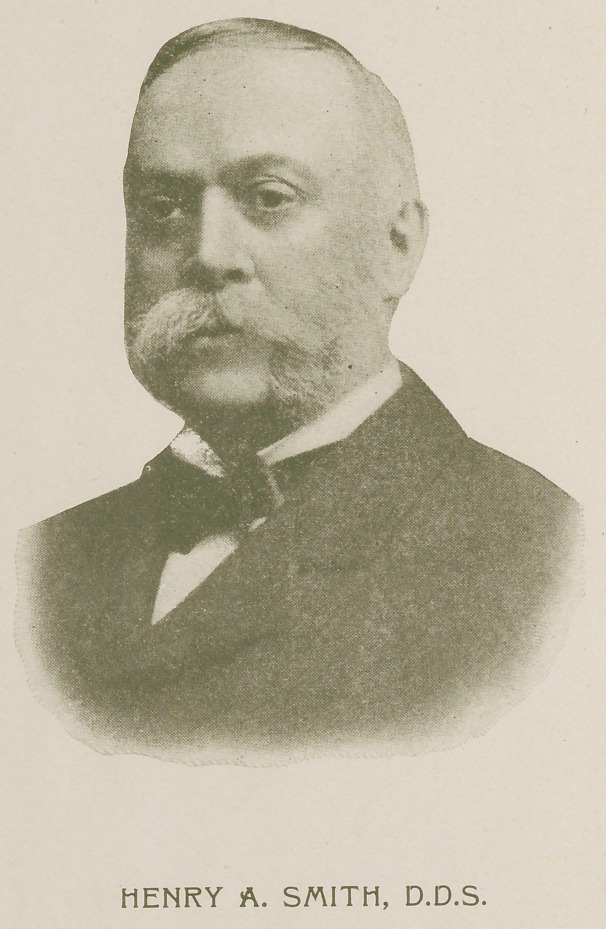# Event and Comment

**Published:** 1907-06-15

**Authors:** 


### THE DENTAL REGISTER.

**Vol. LXI.** **June 15, 1907.** **No. 6.**

### EVENT AND COMMENT.

### Fifty Years in the Practice of Dentistry—Dr. H. A. Smith.

The Dental profession of Cincinnati and vicinity tendered to Dr. Henry A. Smith a banquet at the Queen City Club upon the 50th anniversary of his entering the practice of dentistry. On March 6th, 1857, Dr. Smith was graduated at the Ohio College of Dental Surgery He came to Cincinnati as a young man, from Oxford, Ohio, and entered the office of Dr. Charles Bonsall, a pioneer dentist of this city, eventually succeeding to Dr. Bonsall's practice.

He has seen the profession of Cincinnati grow from ten or a dozen men, with crude appliances and methods of treatment, to the present number of more than 200 dentists.

With every advancement made in the practice and educational fields of dentistry, Dr. Smith has been associated. Every honor in the gift ot the profession has been his. His contributions to its literature and his long experience as a teacher and his constant attendance at its national meetings, has made his name well known to the profession, both in America and abroad. For more than twenty-five years he has been at the head of the Ohio College of Dental Surgery, the pioneer dental school of the territory west of the Allegheny Mountains, and has seen its classes grow from ten students to those numbering nearly 300.

Around the handsomely decorated dinner table March 6th, 1907, loaded with the best that the club could provide, and to music of the orchestra, the following representative denti ts dined, and wished their friend continued health and happiness: Dr. H. A. Smith, Dr. F. Burger, Dr. J. R. Callahan, Dr. J. S. Cassidy, Dr. P. Cassidy, Dr. W. M. Clawson, Dr. C. W. Cragg, Dr. L. E. Custer, Dr. M. H. Fletcher, Dr. W. Foster, Dr. F. Hunter, Dr. J. H. Huschart, Dr. C. I. Keely, Dr. P. K. Phillips, Dr. S. J. Rauh, Dr. A. G. Rose, Dr. R. Siegel, Dr. H. T. Smith, Dr. D. Stern, Dr. D. Stuart, Dr. H. F. Vandervort, Dr. S. G. Walton, Dr. T. I. Way and Dr. C. M. Wright.

Dr. C. M. Wright spoke to the following sentiment "A Man that has Traveled and been careful of His Time:—"*The Antiquary.*" "Generally speaking, a man who travels must be careful ot his time—and his time tables—or he will certainly 'get left.' " A man who has traveled and been careful of his time, manages to accomplish a great deal in his journey through life, and misses idle waiting and failure. He has been a wise man.

Is this the spirit of the toast, or is it that a man who has traveled this fifty years of dental practice and dental teaching, and has arrived at the golden wedding of his career, must have been careful of his time—else how could he have arrived?

Our honored guest, *Dr. Smith,* HAS arrived on time. He therefore has been a careful traveler. He has not missed his train. He has tollowed closely the schedule tor fitty years, since his graduation from the Ohio College ot Dental Surgery, the starting point. He has faithtully traveled along the best lines, lines of patient endeavor, lines ot daily industry, lines ot faithtul service to thousands of grateful patients, lines of earnest study for the advancement of hundreds ot students, lines of indefatigable efforts tor the advancement of the protession of his early choice.

To speak literally after the words of the toast, Dr. Smith *has traveled* tar and wide in this country, and in Europe, and wherever men ot his beloved profession have gathered, and he has been so careful of his time that he has gained inspirations which have sustained him, he has garnered varied opinions, which have been of use to him in his aims, he has acquired a breadth of culture of an enviable reputation throughout the dental world, as a teacher and a practitioner.

What more can a dentist do? What more can we expect oi young men, of our sons, than this? No matter what profession or calling in life they may choose to follow, give a young man just freshly graduated from a professional school—oi law, or medicine, or theology, or dentistry, of architecture or engineering, or any other art or science—what can we wish or hope, or even pray for beyond this, that he shall travel along the route which he has selected, and arrive at the golden anniversary of his trip with a record of having been careful of his time, and at this station to be able to merit the acknowledgment of his confreres that that he has been successful; that he has been an honor to his profession; that the world is better for his having lived and labored, and that his intimate friends may meet around a festive board, and thank him for what he has done, and wish him God speed to the diamond anniversary twenty-five years hence? I do not know of any wider, or higher, or or broader scheme of travel than this. Personally, I have had the honor of having had intimate relations with Dr. Smith for 45 years.

Dr. Smith studied in the office of the lamented Dr. Keeley, of Oxford, Ohio. After spending a year or so as a partner of Dr. Keeley he came to Cincinnati and began his career of practice. "In 1864 I came to Cincinnati, and formed a partnership with Dr. Cameron, after a similar experience with Dr. Keeley So that we were always Keeley's boys; we had the same dental parentage, and from those early days to the present time, we have been intimately associated in many societies, and in the college as teachers. He has always been my superior officer

I need not add to those who know us, and who sit around this table, that I have remained a high private, and am quite likely to miss my train, and wind up in a wreck; but in the meantime, boys, old and young, Here's to the

Captain! Long may he travel, and be careful of his time."

DR. FRANK A. HUNTER, responded to the following toast: "No, never say nothin' without you're compelled to, and then don't say nothin' that you can be held to."

"I presume the reason you call upon me, Mr. Toastmaster, to respond to this toast, is that I am known to be so impulsive that I always say things that get me into trouble. Now, what I would say on this auspicious occasion I am not compelled to, but I am impelled to, and what I shall say I wish to be held to.

Fifty years ago to-night Dr. Smith graduated from the Ohio College of Dental Surgery, and forty years ago to-night I graduated from the Philadelphia Dental College. So you see Dr. Smith and I came very near being twins. Dr. Smith does not care to tell his age, for fear we shall Oslerize him but we know he must be more than a half of a century old, for I am more than fifty myself, and he must be older than I am.

We have watched our honored guest in his honored career, and marked his upward steps. I wish I could tell you younger men how much you are indebted to Dr. Smith for the condition of our profession in this city, making your position in society one of respectability and repute. His life in this city has been such that he has given to our calling a reputation for learning and altruism that has given us a professional standing of no mean order, a benefit that we should covet and never disregard.

Professionally, our friend has practiced conservative dentistry in the best way. He has not been known as an extravagant charger, but has always stood for respectable and suitable fees. No practitioner in this city has had a more loyal clientele; his patients were never of the shopping variety. Because of his persistent stand for his profession, we are all better appreciated and better paid. I want to thank the committee in charge of this most delightful occasion for its thoughtfulness in taking this method of publicly recognizing the work of our friend, and I most sincerely trust that the remaining years of his life may see more of advancement in his beloved profession than he has seen in the fifty eventful years that are gone.

To this sentiment I wish most cordially we all may be held."

Dr. J. S. Cassidy responded as follows to the toast, "What hath this day deserved?"

"I have a feeling somehow, that if Dr. Smith were not present, I would be able to say something really worth while and more or less appropriate to this happy occasion.

It *is* embarassing to say right to a man's face, that he is among the few very best fellows in the world; almost as much so as to call him a vice versa, or some *other* undesirable adjective.

Nevertheless, I take great pleasure, notwithstanding this embarassment, in following Dr. Callahans' instruction by expressing myself just as I please, at least for to-night; and if there be a redundancy of personal pronouns in what I may say, the apology for such is herewith submitted in advance, with the hope that it will cover also all other deficiencies as well.

The many years that almost imperceptibly have drifted with the ebbing tide of time since I first knew of Dr. Smiht have added to the unalloyed appreciation of the privilege ot his acquaintance, and to the honor that I have always felt to be mine in the knowledge of his friendship.

To-night is one well worthy of a private celebration of my own; for memory easily recalls the personality, and the instructive novelty to me, of the commencement exercises of the Ohio Dental College in the first week in March, 1868. It was there I first saw Dr. Smith. He was not a member of the faculty at that time, nor for several years afterwards, but nevertheless he showed his deep interest in the dental education of that day by never failing to attend those annual occasions; and I am willing to assert, even without personal knowledge of those events preceding my own observation, that for fifty years, either as an interested witness or participator, he never once missed the graduation exercises of his Alma Mater. Any man should be proud of such a record. It is unique in the tremenduous possibilities it affords as an object lesson to those who alas are too much disposed to be quitters, who too soon leave the field of life's strenuous race toward success, to those whose staying qualities prove them to be the only exemplars of that magnificent truth, "the survival of the fittest."

During that first week in March, thirty-nine years ago, the Mississippi Valley Dental Association, then in full flower, held its regular session, and although only a junior student, I remained over in order to witness the doings of a dental meeting.

The frisky microbe, and the now ubiquitous bacterium had not yet appeared; but the mallet just then introduced was a sufficient subject for discussion. Needless to say it was ably handled *pro* and *con*, for among the members were Drs. Taylor, Taft, Watt, Morgan, Keeley, Cutler and McKellops, and in that discussion Dr. Smith, though a young man, proved himself the peer of the best. By the way, Dr. Frank Hunter was also there, and I marveled much, not knowing anything of the philosophy of sound, how it was possible that a deep semi-bass voice could come from a body that apparently had no room for either lungs or bronchial tubes.

Permit me to mention a little incident of the meeting which exhibited the characteristic love of justice and fair-play ingrained in the nature of our guest. Some one brought charges of misconduct against a member from the interior of the state. The vote of censure was about to be taken, when Dr. Smith called a halt, and insisted that the accused was entitled to a hearing; the matter was laid on the table, and so the good name ot the absent one was not dishonored.

When a man is master of a private business of legitimate and sufficient scope to engage all his energies in promoting its continuous success, is induced thro' love of a cause, to jeopardise that private business by assuming the leadership thrust upon him ot rehabilitating a loved concern on the verge of utter ruin, thro' circumstances over which no one seems to have control, such a man is deservedly marked as superior to his fellows, and a blessing to the world. Some of you know that our college was at one crucial time in such condition, when Dr. Smith, sacrificing his own personal wishes and comfort, agreed, under protest, to take upon himself the onerous and discouraging task of devising ways and means of rejuvenating the loved old institution, that it indeed might live and become, as it has thro' the force of his determination, one of the foremost of its kind, no longer the plaything of a go-as-you-please mismanagement, no more, as it once was, a suffering, helpless example of the heaven-born truth that he " who gathereth not with me, scattereth."

As to the implied question printed in the menu, the answer is in the fact that we are here to-night to celebrate the golden anniversary of the day that gave Dr. Smith to the profession; to congratulate him, and wish him many, many happy years to enjoy the distinction to which he has attained, honoring ourselves the more by thus honoring him, this good citizen, good dentist and good friend, one to whom her work accomplished, nature bowed in pleased satisfaction, and proclaimed to all the world, "this is a man."

Dr. Smith, in response to the many complimentary speeches, expressed his happiness and appreciation of the cordial and gracious testimonials. He maintained the proposition that the credit for a life spent in the higher development of one's calling was not a selfish one, and when so pursued, it was inevitable that sore disaster would overtake the most industrious worker. He rather sympathized with the sentiment of Dr. Oliver Wendell Holmes, who believed that every man was a debtor to his profession, and is compelled by all the laws of decency to respect and honor it with a loyal devotion to its service in the highest realms.

The beautiful surroundings of the occasion lent enthusiasm and good spirit, and the occasion will long be memorable to all that participated.

## Figures and Tables

**Figure f1:**